# Association between body shape index and small dense LDL particles in a cohort of mediterranean women: findings from Progetto ATENA

**DOI:** 10.3164/jcbn.17-13

**Published:** 2017-08-11

**Authors:** Marco Gentile, Gabriella Iannuzzo, Amalia Mattiello, Fabiana Rubba, Salvatore Panico, Paolo Rubba

**Affiliations:** 1 Department of Clinical Medicine and Surgery, University of Naples "Federico II", Via Pansini 5, 80131 Naples, Italy; 2 Department of Public Health, University of Naples "Federico II", Via Pansini 5, 80131 Naples, Italy

**Keywords:** body shape index, women, small dense LDL

## Abstract

Small dense LDL particles (sd-LDL) and body shape index (ABSI), were evaluated in 228 women, living in Naples, Italy (Progetto ATENA). Serum cholesterol, HDL-cholesterol, LDL-cholesterol, triglycerides, insulin, HOMA, Apo B, hs-CPR and sd-LDL were measured. LDL particle separation was performed by Lipoprint System: seven LDL subfractions were obtained and LDL score (% of sd-LDL particles) calculated. ABSI was calculated according to Krakauer’s formula: ABSI (m^11/6^ kg^−2/3^). The association between sd-LDL and ABSI was evaluated taking into account different adjustment models. Women with elevated levels of ABSI show the following OR of having high LDL score: 2.39, *p* = 0.002; unadjusted; 2.47, *p* = 0.002; adjusted for age; 2.13, *p* = 0.011; adjusted for age and Apo B; 1.93, *p* = 0.026; adjusted for age and Apo B and triglycerides. ABSI was associated with elevated LDL score independently of age, Systolic pressure, Apo B and triglycerides. Median of LDL diameter decreased among ABSI quartiles: quartile I: 271.5 nm, quartile II: 270.7 nm, quartile III 270.5 nm, quartile IV 269.4 nm; Kruskall Wallis Test: *p* = 0.016. These results are in line with the hypothesis that ABSI could be a marker of visceral abdominal associated to adverse metabolic changes including presence of elevated sd-LDL, a risk factor for premature cardiovascular disease.

## Introduction

Small dense LDL particles (sd-LDL) have been highlighted among the biochemical markers associated with metabolic syndrome,^(1)^ carotid atherosclerosis,^(2)^ coronary heart disease^(3)^ and stroke.^(4)^ A number of study have shown that body mass index (BMI), waist circumference (WC), were all associated with increased mortality.^(5–6)^ Recently, a new anthropometric measure, body shape index (ABSI) has been suggested to be superior to body mass index (BMI, kg/m^2^) and WC as a measure of disease risk, in particular for premature mortality, in the general USA population.^(7)^ ABSI could be an accurate marker of visceral abdominal fat compared to peripheral tissue associated to adverse metabolic changes^(7)^ including presence of elevated small dense LDL. In a recent study by Dhana *et al.*^(8)^ ABSI have showed a stronger association with total, cardiovascular and cancer mortality than BMI, WC, waist-to-height ratio (WHtR), waist-to-hip ratio (WHR). Aim of our study was to test the hypothesis that ABSI was associated with high levels of sd-LDL particles in a sample of menopausal women taken from the general population, living in the metropolitan area of Naples, Southern Italy, and participating in a large, ongoing, prospective study (Progetto ATENA study).^(9)^

## Methods

### Study population

The Progetto Atena was carried out on 5,062 women aged 30 to 69 years living in the area of Naples (Southern Italy).^(9)^ The main purpose of this study is to investigate the causes of those chronic diseases that have a major impact on the female population. Potential participants with previous diagnoses of myocardial infarction, stroke, and major cancers were excluded at baseline visit. As a part of the design the recall of 228 women—randomised among the older ones—was scheduled in 2002–2004, after a period of approximately 10 years from baseline. On these participants, all of them in post-menopause, a number of biological and biochemical investigations were performed. Data derived from the biochemical analysis, including small LDL size (period 2002–2004), constituted our sample of observations. No significant differences in cardiovascular risk profile between the subsample with subsequent LDL particle size determination (*n* = 228) and the remaining cohort (*n* = 4,834) were detected.^(2)^ All women gave written informed consent, and the study was approved by the ethical committees of the institutions involved.

### Clinical and biochemical assessment

 Anthropometric measurements were made with the subjects in indoor clothing and without shoes. ABSI was calculated according to Krakauer’s formula: ABSI (m^11/6^ kg^−2/3^).^(7)^ Body mass index, used as a measure of general obesity, was calculated as weight (kg) divided by height (in m^2^). Waist circumference, an index of abdominal obesity, was measured midway between the bottom of the rib cage and the top of the iliac crest. Sitting brachial blood pressure was measured 2 times after a ≥5 min rest using a random-zero sphygmomanometer. A standard questionnaire was used to collect information about smoking habits. Blood specimens were collected after a 12- to 14-h fast, from 8:00 to 9:30 a.m., to reduce the influence of circadian variation.

Total cholesterol, triglyceride and high-density lipoprotein-cholesterol concentrations were measured using enzymatic methods.^(10)^ LDL cholesterol was calculated according to the Friedewald formula. Fasting glucose levels were enzymatically determined by the peroxidase method. Fasting insulin levels were determined by enzyme immunoassay (Ultrasensitive Insulin Elisa, Mercodia, Sweden). The error of the method [between-run coefficient of variation (CV)] was evaluated on the 2 sera at small and large contents of insulin and was <10%. Apolipoprotein B and hs-CRP were measured with turbidimetric assay with an automated method (Cobas-Mira, Roche, Italy). The error of the method (between-run CV) was evaluated by daily analysing a plasma pool and was <5%. The homeostatic assessment model (HOMA) index was used to estimate insulin resistance and calculated as fasting serum insulin (µU/ml) × fasting serum glucose (mM)/22.5, as described by Matthews *et al.*^(11)^

All biochemical analysis were performed on fresh blood sample, only small LDL size were performed on frozen samples (−80°C).

### LDL particle size determination

LDL particles separation was performed by Lipoprint System (Quantimetrix Inc., Redondo Beach, CA). This method^(12)^ is based on electrophoresis of lipid stained serum (Sudan black) in non-denaturing gel gradient of polyacrylamide. Seven LDL subfractions of decreasing size and increasing density and electrophoretic mobility (Rf) are obtained. The coefficients of inter- and intra-assay variation for cholesterol distribution in the 7 LDL species were less than 10% for both. Mean LDL particle diameter (MPD) is calculated on the basis of the different areas under the curve of the 7 LDL species with different electrophoretic mobility.^(8,13,14)^ The proportion of sd-LDL particles (subfractions 3–7) to the whole LDL area (subfractions 1–7) was also calculated in our sample (LDL score) (1). The diameter of the LDL particles at the cut-off point separating subfractions 1–2 from subfractions 3–7 was 25.1 nm. LDL score, which has been shown to be significantly related to coronary heart disease (CHD) in multivariate analysis,^(15–17)^ proved to be significantly related to MPD in the studied population (*n* = 228, Spearman ρ −0.885, *p*<0.001).

### Statistical analysis

Statistical analyses were performed using SPSS version 13.0 (SPSS Inc., Chicago, IL). Continuous variables were described as mean and SD or SE. Logistic regression analysis at univariate and multivariate level multivariate logistic regression analysis was used to test the independent relation between age, BMI, WC, ABSI, Apo B and triglycerides, systolic blood pressure, HDL, Total cholesterol, Smoking habits (independent variables) and LDL score (above 50th percentile LDL score level). Odds ratio (OR) for one unit change of LDL score were calculated by unconditional logistic regression and 95% confidence intervals (CI) of the odds ratio were computed. Correlations coefficient between LDL score and WC or ABSI were calculated by non-parametric Spearman’s ρ.

## Results

Physical and biochemical characteristics of the study participants are shown in Table 1. Out of the 228 participants, 93 (40.8%) met the metabolic syndrome criteria, 77 (35%) used antihypertensive drugs, 26 (12%) used hypocholesterolemic drugs, 62 (30%) were smokers, 78 (40%) were moderate drinkers and 21 (9.2%) met criteria for diabetes. The prevalence of overweight/obesity, according to WHO classification in the population studied, was 71%.

**Fig. 1:**
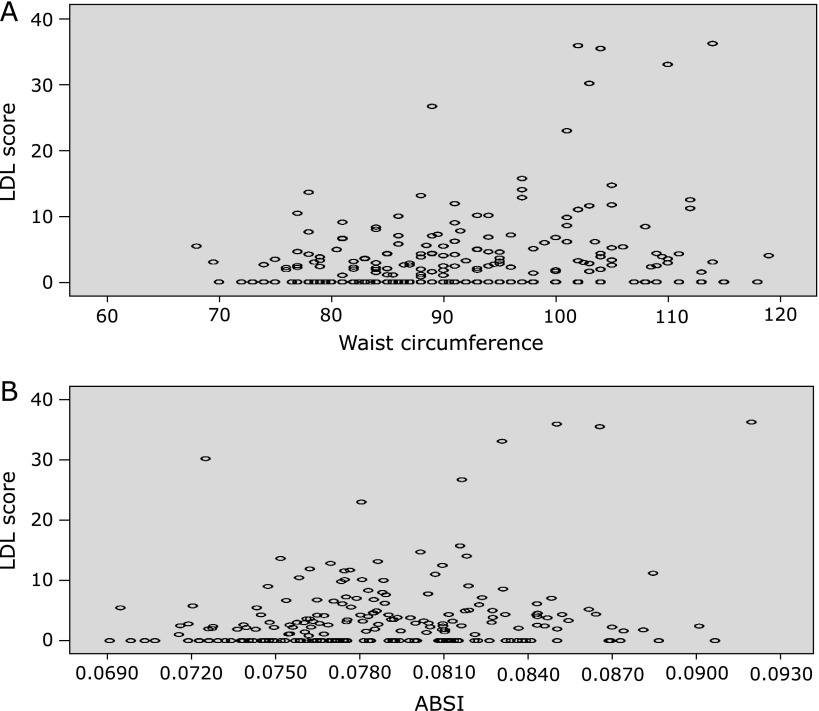
Correlation charts between LDL score and Waist Circumference (WC) or ABSI. LDL score exhibited positive and significant correlation with WC (Spearman’s ρ 0.209, *p* = 0.002) (A) and ABSI (Spearman’s ρ 0.202, *p* = 0.003) (B), (*n* = 228). *N* represents the number of subjects with data available.

Univariate logistic analysis (Table 2) showed a positive and significant relationship of LDL score (above 1.93%, 50° percentile of studied population) with WC (*p* = 0.029) and ABSI (*p* = 0.002), Apo B (*p*<0.001), total cholesterol (*p* = 0.001), triglycerides (*p*<0.001), a negative and significant relationship with HDL (*p* = 0.007) and smoking habits (*p* = 0.026) but not with age (*p* = 0.828), BMI (*p* = 0.537), and systolic blood pressure (*p* = 0.182). Correlation charts between LDL score and WC or ABSI were reported in Fig. 1. Correlations coefficient (Spearman’s ρ) were 0.209 (*p* = 0.002) for WC and 0.202 (*p* = 0.003) for ABSI.

We evaluated the association between LDL score (above 1.93%, 50° percentile of studied population) and WC taking into account different adjustment models (Table 3). After adjustment for age women with WC size (above 50th percentile of the studied population) showed the following OR of having LDL score (above 1.93%, 50th percentile of the studied population): OR = 1.80 (*p* = 0.029). This association resulted not statistically significant after adjustment for age and apo B or age, apo B and triglycerides.

Then, we evaluated the association between LDL score (above 1.93%, 50th percentile of the studied population) and ABSI taking into account different adjustment models. Univariate logistic analysis showed a positive and significant relationship of ABSI (above 50th percentile of studied population), with systolic blood pressure (*p* = 0.003), triglycerides (*p* = 0.003), apo B (*p* = 0.003), age (*p*<0.001) and total cholesterol (*p* = 0.005), but not with HDL (*p* = 0.166) and smoking habits (*p* = 0.755). Women with ABSI (above 50th percentile of studied population), showed the following OR of having LDL score (above 1.93%, 50th percentile of studied population): 2.47 (*p* = 0.002), adjusted for age; 2.13 (*p* = 0.011), adjusted for age and apo B; 1.93 (*p* = 0.026), adjusted for age, apo B, systolic blood pressure and triglycerides (Table 4). This relation was further confirmed in multivariate analysis after the following adjustments: OR 2.43 (*p* = 0.003), adjusted for age and use of antihypertensive drugs; OR 2.56 (*p* = 0.002), adjusted for age and use of hypocholesterolemic drugs; OR 2.52 (*p* = 0.002), adjusted for age and smoking habits; OR 2.38 (*p* = 0.004), adjusted for age and alcohol consumption; OR 2.32 (*p* = 0.004), adjusted for age and diabetes diagnosis.

**Fig. 2:**
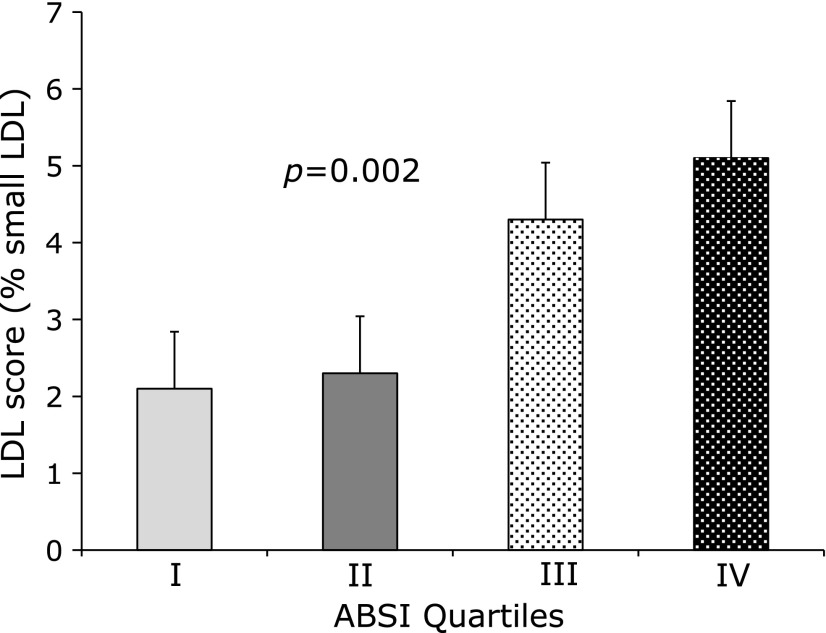
Relation between LDL score and quartile of ABSI in the studied population (*n* = 228). All Values are Means ± SE. *N* represents the number of subjects with data available. *P* value between ABSI quartiles are obtained by non-parametric Kruskal-Wallis test.

Women with elevated ABSI (III and IV quartile of studied population) showed elevated level of LDL score (I quartile: 2.1%; II quartile 2.3%; III quartile 4.3%; IV quartile 5.1%) (*p* = 0.002 Kruskal Wallis) (Fig. 2). Median of LDL diameter decreased progressively among ABSI quartiles: quartile I: 271.5 nm, quartile II: 270.7 nm, quartile III 270.5 nm, quartile IV 269.4 nm; Kruskall Wallis Test: *p* = 0.016.

## Discussion

The main finding of the present study is the association between ABSI (above 50th percentile of studied population) and LDL score (above 1.93%, 50th percentile of studied population) an established marker of cardiovascular risk.^(2)^ The association between ABSI and LDL score is independent of age, Apo B, Triglycerides, in this group of menopausal women. This association can be useful to plan epidemiological trials investigating lifestyle aimed at lowering ABSI levels. The strength of this study is that no data are available on the relationship between ABSI and small dense LDL in general population and in particular in women. A major limitation of the study is that the design is cross-sectional, so we can only detect the association between small dense LDL and ABSI. In a previous report, LDL score, which is the proportion of small LDL particles (subfractions 3–7) to the whole LDL area (subfractions 1–7), has been shown to be significantly related to coronary heart disease in multivariate analysis.^(15–17)^ The presence of sd-LDL particles is associated with two to three fold increase in risk of coronary heart disease.^(5)^ An increased cardiovascular risk characterizes the postmenopausal phase in women.^(18)^ Post-menopausal women have been found to have higher LDL and higher percentage of small dense LDL, despite similar BMI and HDL cholesterol as compared to premenopausal women.^(19)^ The presence of sd-LDL particles is associated with two to three fold increase in risk of coronary heart disease.^(5)^

High ABSI indicates that WC is higher than expected for a given height and weight and corresponds to a more central concentration of body volume and is a risk factor for premature mortality in the general USA population. ABSI expresses the excess risk from abdominal fat in a convenient form that is complementary to BMI and to other known risk factors.^(7)^ A paper by He and Chen^(20)^ demonstrated that ABSI is an independent risk factor to develop diabetes mellitus in the Chinese population. Recently Dhana *et al.*^(8)^ have demonstrated in a prospective population-based study, Rotterdam Study, (*n* = 6,366), 22 years of follow-up, that ABSI was more strongly associated with total cardiovascular and cancer mortality than BMI, WC, WHtR, WHR.

Our study conducted in a sample of postmenopausal women who have participated into a population-based cohort study, living in the metropolitan area of Naples, Southern Italy (Progetto ATENA) demonstrates that the association of sd-LDL with higher ABSI is independent of age, apo B and triglycerides concentration. No similar association was found between BMI or WC and sd-LDL.

These results are in line with the hypothesis that ABSI may be a more accurate marker of visceral abdominal fat which is associated with adverse metabolic changes including presence of elevated small dense LDL, an established risk marker for cardiovascular disease. ABSI may be a useful tool to give additional information in the risk assessment for atherosclerotic disease, in particular in postmenopausal women.

## Figures and Tables

**Table 1:** Clinical and biochemical characteristics of studied population

Variable	*n* = 228
Age (years)	63.1 ± 8.1
Total cholesterol (mg/dl)	224.5 ± 38.4
Triglycerides (mg/dl)	110.9 ± 56.6
High-density lipoprotein cholesterol (mg/dl)	57.8 ± 13.5
Low-density lipoprotein cholesterol (mg/dl)	144.5 ± 34.4
Fasting glucose (mg/dl)	105.1 ± 25.7
Apolipoprotein B (g/L)	1.1 ± 0.2
High sensitive CRP (mg/L)	2.7 ± 4.1
Insulin (mU/L)	6.8 ± 4.2
Body mass index (kg/m^2^)	28.1 ± 4.6
Waist circumference (cm)	91.4 ± 11.1
Homeostatic assessment model index (HOMA)	1.8 ± 1.3
Systolic blood pressure (mmHg)	143.0 ± 21.2
Diastolic blood pressure (mmHg)	81.5 ± 8.8
Mean LDL size (nm)	27.1 ± 0.3
LDL score (% small LDL)	3.6 ± 6.1
Intima-media thickness (mm)	1.0 ± 0.2
ABSI	0.077 ± 0.004

Values are expressed as mean ± SD. International system conversion factors: to convert triglycerides to millimoles per liter, multiply by 0.0113; to convert high-density lipoprotein cholesterol to millimoles per liter, multiply by 0.02586; to convert glucose to millimoles per liter, multiply by 0.05551; to convert total and low-density lipoprotein cholesterol to millimoles per liter, multiply by 0.02586.

**Table 2:** Relationship among LDL score, Age, BMI, WC, and ABSI. Univariate logistic analysis

Independent variables	Dependent variable LDL score*		
	*p*	OR	95.0% CI for OR
			lower–upper
Age (1)	0.828	1.00	0.97–1.03
BMI (1)	0.537	1.01	0.96–1.07
WC (2)	0.029	1.80	1.06–3.06
ABSI (2)	0.002	2.39	1.37–4.16
Apo B (1)	<0.001	11.65	3.17–42.77
Total Cholesterol (1)	0.001	1.01	1.00–1.02
Triglycerides (1)	<0.001	1.01	1.00–1.02
HDL (1)	0.007	0.97	0.95–0.99
Smoking habits (3)	0.026	0.50	0.27–0.92
Systolic blood pressure (1)	0.182	1.00	0.99–1.02

(1) Continuous variables. (2) Discrete variable (above 50th percentile of the studied population). (3) Discrete variable (smoking habits yes/not). *Discrete variable (above 50th percentile of the studied population).

**Table 3:** Relationships between LDL score, WC and other variables (*n* = 228): multivariate logistic analysis

Predictive variables	Dependent variable LDL score*		
	*p*	OR	95.0% CI for OR
			lower–upper
WC (1)	0.029	1.80	1.06–3.05
Age (2)	0.935	0.93	0.97–1.03

WC (1)	0.091	1.60	0.92–2.76
Age (2)	0.853	0.99	0.96–1.03
Apo B (2)	0.001	9.90	2.64–37.10

WC (1)	0.525	1.21	0.67–2.18
Age (2)	0.352	0.98	0.94–1.01
Apo B (2)	0.124	3.20	0.72–14.12
Triglycerides (2)	<0.001	1.01	1.00–1.02

(1) Discrete variables. (2) Continuous variables. *Discrete variable (above 50th percentile of the studied population).

**Table 4:** Relationships between LDL score, ABSI and other variables (*n* = 228): Multivariate logistic analysis

Predictive variables	Dependent variable LDL score*		
	*p*	OR	95.0% CI for OR
			lower–upper
ABSI (1)	0.002	2.47	1.39–4.37
Age (2)	0.649	0.99	0.95–1.02

ABSI (1)	0.011	2.13	1.18–3.83
Age (2)	0.577	0.99	0.95–1.02
Apo B (2)	0.002	8.13	2.12–31.11

ABSI (1)	0.026	1.93	1.04–3.59
Age (2)	0.222	0.97	0.94–1.01
Apo B (2)	0.213	2.60	0.57–11.77
Triglycerides (2)	<0.001	1.01	1.00–1.02

(1) Discrete variables. (2) Continuous variables. *Discrete variable (above 50th percentile of the studied population).
